# Patients’ perspectives regarding antibiotic treatment for acute sinusitis in Norwegian general practice. A qualitative interview study

**DOI:** 10.1080/02813432.2025.2498510

**Published:** 2025-05-07

**Authors:** Jorunn Thaulow, Torunn Bjerve Eide, Sigurd Høye, Holgeir Skjeie

**Affiliations:** ^a^ Department of General Practice, Institute of Health and Society, Antibiotic Centre for Primary Care, University of Oslo, Oslo, Norway; ^b^ General Practice Research Unit, Institute of Health and Society, University of Oslo, Oslo, Norway

**Keywords:** Sinusitis, qualitative research, individual interviews, general practice, antibiotics, patients’ expectations, trust in GP

## Abstract

**Background:**

The prescription rate for antibiotics for acute sinusitis in primary care remains high, despite evidence showing their limited effectiveness. GPs frequently encounter patient demands for antibiotics, which can influence their decision-making process.

**Aim:**

This study aimed to explore the help-seeking and expectations of patients experiencing symptoms of acute sinusitis, with a particular focus on their desire for antibiotics. We sought to understand why patients seek their GP for sinusitis, what kind of treatment they hope to receive, and how they perceive their influence on the GP’s treatment decision.

**Methods:**

We conducted 12 semi-structured interviews with patients who had consulted their GP for acute sinusitis from various regions across Norway. The qualitative analysis was performed using systematic text condensation.

**Results:**

We developed 3 main themes:1. ‘Pain and exhaustion’- Patients primarily consulted their GP for acute sinusitis seeking medication to alleviate pain and accelerate recovery.2. ‘I trust the GP, but mainly myself’- While patients expressed trust in their GPs, they also had clear expectations of receiving the specific treatment they sought. 3. ‘Antibiotics works’- Most patients associated medication with antibiotics and believed they were effective against sinusitis.

**Conclusion:**

Patients with sinusitis primarily visited their GP to seek relief from pain and to obtain medication that they believe could speed up recovery, which often meant antibiotics. These findings suggest a continuing need for measures targeting both doctors and patients to align treatment expectations, improve adherence to clinical guidelines, and adjust patient expectations. Better pain management of sinusitis should be emphasized.

## KEY POINTS

- Sinusitis is a self-limiting condition with infrequent complications, and most patients gain no benefit from antibiotics.
- In our study, patients primarily consulted their GP for acute sinusitis to seek medication for alleviating pain and accelerate recovery.
- Most of the participating patients associated sinusitis treatment with antibiotics and believed them to be effective for this condition.

## Background

Acute sinusitis (AS) is a commonly encountered infection in general practice. Acute bacterial sinusitis is a rare condition, with an estimated incidence of 0.5–2% of viral acute rhinosinusitis (ARS) cases (common cold), as noted in the European Position Paper on Rhinosinusitis and Nasal Polyps 2020 [1]. However, the reported prevalence of bacterial infections in ARS varies widely depending on the population studied and the diagnostic methods applied [2]. Recent studies have shown that antibiotics provide little benefit in treating uncomplicated sinusitis, regardless of its cause. A 2018 systematic review from the Cochrane Database concluded that antibiotics should not be used in the management of uncomplicated acute sinusitis [3]. Considering the growing global challenge of antimicrobial resistance (AMR), it is crucial to understand the factors contributing to the continued overuse of antibiotics [4]. Despite the low incidence of bacterial infection, antibiotics are prescribed for about 50% of patients presenting with acute sinusitis in Norwegian, German, and Canadian primary care settings [5–7]. Research from several other countries confirms that over-prescription of antibiotics for acute sinusitis remains a significant issue [8–12]. For instance, a 2018 British study found that 82% of patients received antibiotics for AS, whereas the ideal prescribing rate should have been 11% [10].

This overprescribing may be driven by exaggerated belief among patients that antibiotics are necessary. A survey-based study in the USA revealed that 70% of patients believed antibiotics were needed for acute sinusitis [13]. At the same time, GPs tend to overestimate patient pressure to prescribe antibiotics, and overprescription may be linked to a lack of patient-centered care [14]. The complex interaction between doctor and patient can contribute to the continuation of inappropriate prescribing [15]. Improved communication, along with providing information about the viral etiology and the ineffectiveness and side effects of antibiotics, may reduce inappropriate expectations for antibiotics [16,17]. Such information has been developed through EPOS4 (European Position Paper on Rhinosinusitis and Nasal Polyps), aiming to help patients be well-informed and prepared to make treatment decisions with their doctor [1,18].

The Norwegian guideline for antibiotic use in primary care recommends antibiotics only if severe symptoms have lasted more than seven days, with phenoxymethylpenicillin as the primary choice for acute sinusitis [19]. In a recent study, we investigated the factors influencing GPs decisions to prescribe antibiotics for patients with acute sinusitis. The findings revealed considerable variability in the management of acute sinusitis among GPs, with many not adhering to the Norwegian guidelines, and opting for alternatives to penicillin when prescribing antibiotics. Additionally, the study identified patient-reported pain and exhaustion as reasons for prescribing antibiotics. Pragmatic considerations, such as the day of the week, stress, and patients travel plans, also influenced the decision-making process [20].

Based on this, we hypothesized that the decision-making regarding antibiotic use for acute sinusitis is complex and influenced significantly by the relationship and communication between doctor and patient. The aim of this study was therefore to investigate what drives patients to visit their GP when they suspect they have acute sinusitis, their thoughts, and expectations regarding treatment, and how they perceive their influence on GPs’ decisions to prescribe antibiotics.

## Materials and methods

### Data collection

We conducted a qualitative study using individual interviews with patients from primary care. This method was selected to gain deeper insight into the reasons behind patient’s actions and expectations. GPs were recruited to the study by the Norwegian Primary Care Research Network (PraksisNett), a national research network for general practice in Norway. GPs affiliated with this network and based in three different cities in Norway were asked to identify patients who had sought treatment for acute sinusitis within the past five years. The cities were chosen to ensure geographical diversity and to capture a range of urban and rural environments. During the recruitment process, GPs were instructed to select patients who had been in contact with them due to acute sinusitis within the past five years and who were willing to participate in an interview.

We performed 12 interviews. Each lasted between 30 and 40 min and were digitally recorded. JT served as the moderator for all interviews. Nine interviews took place at the patients’ GP offices, while the remaining three were conducted *via* video conference due to the patient’s inability to visit the GP office in person.

We used a semi structured interview guide with open ended questions, based on a literature review and discussions within the research group. The BASIC (Better treatment for Acute Sinusitis in Primary Care) project, of which this study is a part, has established a user group (UG) consisting of patients, GPs, and representatives from health authorities. We also consulted the UG when developing the interview guide, receiving valuable feedback. After the first seven interviews, we conducted a preliminary analysis, which led to modifications in the interview guide. The focus shifted from general questions about sinusitis and its treatment to a more specific focus on antibiotic use. The final interview guide is provided in the appendix. Prior to analysis, we excluded two of the initial seven interviews, as neither patient could recall seeking medical attention for upper respiratory infection symptoms. Throughout the study, we use the term acute sinusitis in alignment with the terminology commonly applied in primary care settings and the ICPC-2 coding system (International Classification of Primary Care), which reflects how GPs typically document and discuss these cases. While rhinosinusitis is used in the European Position Paper on Rhinosinusitis, our choice of terminology reflects the clinical and practical context of the study.

### Analysis

JT transcribed all the interviews verbatim. JT, HS and TBE conducted the analysis using Malteruds method of systematic text condensation, a methodical and rigorous approach for conducting thematic cross-case analysis of qualitative data [21]. Initially, all authors read the first seven interviews to gain an overall impression, and during our first meeting, we conducted a preliminary analysis together, resulting in the creation of an initial code tree with preliminary themes. The next steps of the analysis were performed after all 12 interviews had been completed. We adjusted the preliminary themes and developed a new code tree based on the content of all interviews. Together, we selected codes, each represented by meaning units that focused on the patient’s perspective on treatment considerations. SH independently read the interviews and provided feedback on the themes and coding. In the final part of the analysis, we created condensates representing the essence of each theme. Finally, we conceptualized the entire content as a synthesis reflecting the patient’s perspectives on sinusitis treatment. All authors reached a consensus on the final developed themes, subthemes, and the illustrative quotes.

JT is a specialist in ear, nose, and throat (ENT) care, while HS and TBE are specialists in family medicine. SH is a general practitioner, the leader of the Antibiotic Centre for Primary Care, and the medical editor of the National guidelines for antibiotics in primary care. All four authors are researchers at The Department of General Practice, University of Oslo, and are affiliated with the Antibiotic Centre for Primary Care (ASP), a national center of competence under The Department of General Practice. As GPs and ENT specialists, our clinical experience provides valuable insight into the complexities of GP-patient decision-making, including the balance between patient expectations and evidence-based practice. Together, we have reflected on and accounted for our preconceptions during the analysis to ensure that our interpretations remain grounded in the study data. The quotes in this study were translated from Norwegian to English by the authors. Data was conducted using NVivo12 software, and the reporting adhered to the Consolidated criteria for Reporting Qualitative studies (COREQ) [22]. After completing the manuscript, we used GPT UiO version GPT-4 Omni for language editing, ensuring that the original intent of the text was preserved [23]. GPT UiO is an implementation of OpenAI’s GPT-4 Omni, specifically adapted and hosted for use by the University of Oslo (UiO), with access restricted to UiO employees only.

## Ethics

The Norwegian Centre for Research Data approved the data protection measures for this study (ref.no. 856919). As the study did not involve any sensitive patient information or interventions (ref.no. 98690), approval from the Regional Committee for Medical and Health Research Ethics was not required. All participants provided informed consent to participate in the study. To ensure confidentiality, any sensitive information was redacted from the transcriptions, and non-identifiable designations were used in the quotes.

## Results

The characteristics of the participants are presented in Table 1. The primary reasons given for contacting a GP when experiencing symptoms of acute sinusitis were to receive medication for pain relief and/or to speed up recovery. Many participants expected to be prescribed antibiotics, believing them to be the most effective treatment. The majority expressed trust in their GPs’ clinical judgement and advice. The analysis of the interviews identified three main themes:

**Table 1.** Characteristics of the participants.

Total number of participants	12
Median age (range)	41 (18–60)
Women	6
Men	6

1. ‘Pain and exhaustion’- Patients primarily consulted their GP for acute sinusitis seeking medication to alleviate pain and accelerate recovery.
2. ‘I trust the GP, but mainly myself’- While patients expressed trust in their GPs, they also had clear expectations of receiving the specific treatment they sought.
3. ‘Antibiotics works’- Most patients associated medication with antibiotics and believed they were effective against sinusitis.

### Pain and exhaustion

#### Expecting antibiotics

Many patients were confident in their ability to recognize when they had a severe sinus infection. These patients often tried various home remedies and delayed visiting their GP for as long as possible. Eventually, they felt that the only remaining solution was to consult the doctor for medication. Consequently, by the time they visited the doctor’s office, they had clear expectations of receiving a prescription for antibiotics. This expectation was frequently cited by participants as the primary motivation for seeking a consultation with their GP.

I expect him to give me antibiotics, to help me, and not just let it go on for three more days. Because if not, I don’t know what I would do, three more days, it’s like three times worse. (Participant nr. 9)

#### General condition

Several patients considered their general condition to be a significant factor in deciding whether to consult their GP for acute sinusitis. As their infection worsened and began to impact their ability to work or function normally, the threshold for seeking medical advice decreased. Some individuals mentioned that they could still manage to go to work but struggled to maintain optimal functioning, experiencing fatigue and reduced productivity. They also described feeling exhausted due to sinus pressure, which led to disrupted sleep and increasing tiredness over time.

The decisive factor is, of course, the general condition, how I feel in my body, that I feel sick, because it can manifest in a way that…. I feel unwell, and in pain. (Participant nr. 3)

#### Pain

The pain and pressure caused by sinusitis were frequently identified by many participants as the main issues and the primary motivation for seeking help from their GP. Their concerns about the pain were expressed in various ways, but there was a unison belief that they needed their doctor’s assistance to manage such intense discomfort. They sought medication to alleviate the pain and pressure, with many participants believing that antibiotics would be effective in achieving this. Additionally, several participants mentioned that they thought they needed anti-inflammatory medication, mistakenly believing that antibiotics would provide the desired effect. This suggests some confusion between the terms anti-inflammatory and anti-bacterial medication.

If I bend down to tie my shoes and it feels like someone is standing on my head, that’s when I realize that it may not be pleasant to walk around being so congested, and I usually get some penicillin. (Participant nr. 5)

When the pain becomes so intense that I can no longer ignore it, I know I need to take action. When it gets so uncomfortable that it really impairs my daily life, I see my doctor. (Participant nr. 2)

#### Duration

Several patients acknowledged that they would likely recover if they waited long enough, but they also expressed a lack of time or patience to wait for this to happen. Some were also concerned that their condition might worsen if they delayed seeking treatment. The timeframe within which participants decided to contact their GP varied between three and fourteen days after the onset of symptoms.

It took me a while to go to the doctor, since I was used to having headaches. Eventually, I became very frustrated after maybe nine days, I realized it was constant, it wouldn’t go away at all. Painkillers didn’t work either, so I finally decided to see the doctor. (Participant nr. 12)

### I trust the GP, but mainly myself

#### Patient-driven treatment

Several patients were confident in their ability to self-diagnose sinusitis. Having experienced it before, they were familiar with the symptoms and believed that booking a consultation with their GP was a waste of time, as they typically only needed a prescription for antibiotics.

I already know the answer before I even go, it’s just that I can’t sign the papers myself. (Participant nr. 7)

It’s the same thing every time. I get 10 days of penicillin, and then it goes away. Then after six months, I need another 10 days and it’s gone. I don’t think blood tests will show anything different than they always do: ‘Now you have a sinus infection, now it’s gone. (Participant nr. 5)

#### Trust in their GP’s judgement

Many of the patients exhibited a significant level of trust in their GP and had confidence in the decisions made during consultations. This trust was often reinforced when they underwent a physical examination, often supplemented by diagnostic tests. Several patients believed that tests like C-reactive protein (CRP) were necessary to accurately diagnose their condition and determine the best treatment. Even patients who were confident in their own diagnostic abilities still trusted their GP, but they expected their GP to agree with their assessment.

I can’t say with one hundred percent certainty without having seen the doctor whether it’s sinusitis, a regular cold, or a combination of both. (Participant nr. 3)

### Antibiotics work

#### Antibiotics work on pain

One of the most bothersome complaints reported by the participants was the pain and the pressure caused by sinusitis. Many believed that antibiotics were the only effective medication to alleviate this pain and relieve the pressure. Some participants also expressed fear of what would happen if they were denied antibiotic treatment.

I’m afraid the authorities will decide, ‘we can no longer provide antibiotics to people with sinusitis’, which would be unfortunate for those who haven’t had surgery or cannot undergo surgery. It would mean no improvement, and they would be left to suffer. It’s really a torturous feeling. (Participant nr. 9)

#### Antibiotics affect the duration

Several patients believed that antibiotics would shorten the duration of their illness, allowing them to recover quickly and return to work or resume their daily activities sooner. Some acknowledged that they might have recovered after a few weeks if they had simply waited, but they didn’t have the time or patience to do so.

I guess it probably would have gone away on its own, but the antibiotics helped clear it up faster. (Participant nr. 12)

#### Antibiotics work according to previous experiences

Belief in the effectiveness of antibiotics was often rooted in personal experiences. Participants referred to past episodes where they had recovered after taking antibiotics or instances where their condition failed to improve when they waited too long. Those who had multiple positive experiences with antibiotics for sinusitis were particularly convinced of the necessity of receiving antibiotics whenever they experienced symptoms of sinusitis.

I just think that the diagnosis itself should be enough for the doctor to recommend antibiotics. Based on my experience, I believe it would be the best option. (Participant nr. 1)

I’m getting older, and I know that if it’s a sinus issue, if I feel that familiar sensation, then I know antibiotics are what will work. (Participant nr. 7)

#### Antibiotics prevent the infection from spreading

Some patients believed that antibiotics were crucial in preventing the infection from progressing to a more severe stage. Several indicated that if they noticed the pressure spreading to new areas of the face or towards the ears, their concern increased. These patients shared the belief that antibiotics could prevent such disease progression.

So, it can certainly happen that one can develop a severe infection over time, in the brain or somewhere inside the head. (Participant nr. 11)

## Discussion

### Summary of main results

Many patients expressed trust in their GP and valued their expertise, especially when the GP conducted a thorough examination and used diagnostic tests, such as CRP (C-reactive protein) or nasopharyngeal culture, to confirm the diagnosis. Some patients, however, were confident in their ability to self-diagnose sinusitis and believed that scheduling a GP appointment was unnecessary, as they felt they only needed antibiotics. They trusted their own judgment and had a predetermined expectation of the treatment they should receive. Patients who were confident in their own diagnosis also expected their GP to share their opinion.

Many patients believed that antibiotics were essential for managing the pain and pressure caused by sinusitis. They were concerned about the consequences of being denied antibiotic treatment, believing that antibiotics would shorten the duration of their illness and allow them to resume their daily activities sooner. This belief in the effectiveness of antibiotics was often based on past experiences where antibiotics had helped them recover from sinusitis or prevented the infection from progressing to a more severe stage.

#### Comparison with existing literature

Our findings suggest that patients are more likely to trust their GPs when they receive a comprehensive examination and diagnostic tests, which help them feel taken seriously. Previous research has shown that factors such as ‘being taken seriously’ are strongly associated with trust in GPs [24]. When patients perceive their GP as conducting thorough examinations and addressing their concerns, their trust increases. This situation rises several important issues, including the possibility that GPs may feel compelled to perform unnecessary tests to build trust and the potential pressure to prescribe antibiotics for the same reason. Several studies indicate that GPs may overestimate the pressure to prescribe antibiotics or may do so to maintain patient relationships, with trust playing a crucial role for both parties [14,25,26].

Kleinman’s theory of explanatory models focuses on how individuals understand illness and health challenges through the perspectives shaped by their social, cultural, and personal backgrounds [27]. This framework clarifies the different ways in which individuals perceive and respond to health issues. Our study indicates that patients frequently have definitive views on when their conditions warrant antibiotics, often guided by their pain levels, which they associate with a need for medical intervention. These patient-derived explanatory models, emphasizing personal experiences of symptoms, contrast sharply with those of doctors, who may attribute the pain to pressure-related causes rather than a bacterial infection necessitating antibiotics. Kleinman describes that conflicts between patient and doctor can arise because they talk past each other without noticing. In this way, discrepancies between the explanatory models used by the doctor and the patient to interpret the symptoms develop and intensify. The divergence between these models can lead to challenges in clinical settings, particularly when differences in understanding affect doctor-patient communication and when there is professional uncertainty regarding treatment options, such as in cases of sinusitis [20]. These dynamics highlight the critical need to bridge the gap between patient and professional perspectives to improve healthcare outcomes.

A recent qualitative study examining patients’ perspectives on the drivers and deterrents of antibiotic treatment of acute sinusitis found that patients often hold misconceptions about the indications and effectiveness of antibiotics for AS [28]. The research identifies several drivers, including the belief that antibiotics are a convenient and/or effective way to relieve or cure sinusitis, particularly in addressing discolored rhinorrhea and providing symptom relief. Additionally, patients were motivated by the desire for tangible outcomes after a doctor’s visit [28]. These findings align with the main results from our study, although the emphasis on pain is even more pronounced in our findings, where many patients described that the need for treatment was obvious due to the severity of their pain. Our results also indicate that many patients are confident in their ability to self-diagnose and determine the need for antibiotics, requiring only the doctor’s authorization to proceed.

Our results clearly show that patients want to be actively involved in the decision-making process regarding the choice of treatment, they desire thorough examinations, and they want to be taken seriously. Altiner et al. concluded in their 2004 study that there was a lack of patient-centeredness in decisions regarding antibiotic prescriptions, and our findings suggest that this issue remains unresolved [14]. We recognize that changing ingrained habits around the doctor-patient relationship and treatment decisions can be challenging [29,30].

Our results demonstrate that many patients already expected antibiotics to be the most effective treatment even before the consultation. A study based on video recordings of numerous consultations found that anticipating and communicating a non-antibiotic outcome can help counter patient pressure. Additionally, the timing and manner of communication about antibiotics during the consultation are crucial [15]. Based on our findings, it is important to consider ways to lower patient expectations of antibiotics even before the appointment. Established triage systems, however, may overestimate the severity of transient infections [31]. Tools based on artificial intelligence (AI) show promising results in triaging patients with upper airway symptoms, potentially identifying low-risk cases that can be managed without face-to-face consultations. This approach could reduce unnecessary antibiotic prescriptions and improve healthcare efficiency [32,33].

In the 1997 study by Little et al. on prescribing strategies for sore throat, the authors concluded that both current and previous prescribing of antibiotics for sore throat increase the likelihood of patient reattendance, illustrating the medicalizing effect of such prescribing practices [34] Our findings align with this and shed light on how GPs’ willingness to accommodate patients’ requests for antibiotics contributes to initiating and sustaining a cycle of medicalization.

Moreover, our findings suggest that patients’ responses may reflect a medicalizing effect caused by earlier antibiotic prescribing, particularly for conditions like sinusitis. This aligns with observations from our 2023 publication, where GPs described some patients as ‘demanding’ or ‘pushy’ in seeking antibiotics for sinusitis symptoms, attributing their prescribing decisions to these patient behaviours. However, the present study indicates that this dynamic is, in part, created by the GPs’ own prescribing patterns, which establish expectations among patients and encourage repeated requests for antibiotics.

The interplay between patient behaviour, GP prescribing patterns, and the resulting expectations highlights the critical role of GPs in breaking this cycle. Explicitly addressing the medicalizing effect of antibiotic prescribing in both clinical practice and public health strategies could help shift this dynamic toward more sustainable prescribing practices.

#### Methodological considerations

The interviews were conducted over a three-year period across three different regions of Norway, varying in urbanization and population size. The interview period extended beyond the initial plan due to the Covid 19 pandemic. We interviewed 12 participants, aged 18 to 60 years, with an equal number of men and women. This diversity enhanced the study’s external validity, and the variation in age and gender contributed to a rich and varied dataset. We chose to include consultations from the past five years to ensure that GPs could identify enough eligible patients, acknowledging the potential for recall bias. While some participants had limited recollection of their consultations, this could also be an advantage, as some had experienced multiple episodes for comparison or developed deeper reflections on the topic.

Although this was a cross-case analysis with a small, specific subject, the depth of the dialogue and the number of participants provided sufficient information power, as outlined by Malterud [35]. Internal validity in a study like ours will be challenged by response bias, where participants might tell the interviewer what they think the interviewer wants to hear, – as well as confirmation bias where the data is interpreted to fit the researchers’ preconceptions. While we cannot entirely exclude these biases, we took great care to maintain the participants authentic voices through open dialogue interviews and critical discussions during the analysis.

The lead author, who has experience as an ENT specialist, municipal superintendent, and general practitioner, brings a diverse perspective to the study. However, this background may have introduced certain biases and influenced her understanding of the working conditions and challenges faced by general practitioners, potentially affecting her interpretation of the data and insights drawn from the patient interviews. Acknowledging this potential bias, the three co-authors, all experienced general practitioners, contributed to balancing the discussion. While working on the results from a previous focus group study on general practitioners’ reasons for prescribing antibiotics for sinusitis, in which she also served as the lead author, she developed a strong interest in AI [20]. This interest led her to explore how this emerging technology could help reduce antibiotic use and promote treatments more aligned with clinical guidelines. She also began to investigate and develop tools based on generative AI to assist general practitioners in making better-informed decisions.

The insights she gained in this field likely influenced some of the ideas presented in the discussion section. However, the growing focus on artificial intelligence and its broader application in healthcare is a significant and rapidly evolving trend, making it highly relevant in today’s context.

## Implications

These findings highlight the importance of both reducing unnecessary doctor visits through better patient education focusing on self-care and appropriate treatment options, as well as empowering GPs to break the cycle of medicalization by providing clear guidance, managing patient expectations, and promoting sustainable prescribing practices. Adequate pain management are considered important in this context. Our results suggest that GPs have an opportunity to leverage patients’ trust by adopting stricter criteria for prescribing antibiotics while offering alternative pain relief and decongestants more liberally. Conducting a simple physical examination, even if it doesn’t seem necessary from the doctor’s perspective, can significantly impact the patient’s sense of being taken seriously. If patients are advised on pain relief medication and decongestants as an alternative to antibiotic treatment, we believe that antibiotic prescriptions could be significantly reduced.

## Appendix

### Topic guide for individual patient interviews

1. How would you describe “sinusitis”?
2. What would make you think about calling your doctor if you had symptoms of sinusitis?
3. How do you think your doctor can help when you have sinusitis?
4. How much do you trust the advice or treatment you get from your doctor?
5. What are your thoughts on using antibiotics for sinusitis?
6. How do you think doctors figure out if the infection is from a virus or bacteria?
7. Are there specific symptoms that make you feel you need antibiotics more than others?
8. What do you think might happen if you didn’t get antibiotics for sinusitis?
